# Impact of the universal health insurance benefits on cervical cancer mortality in Colombia

**DOI:** 10.1186/s12913-024-10979-0

**Published:** 2024-05-31

**Authors:** Almira G.C Lewis, Diana M. Hernandez, Isabel C. Garcés-Palacio, Amr S. Soliman

**Affiliations:** 1 Department of Global Health, Boston University School of Public Health, Boston University, Boston, MA USA; 2 Department of Sociomedical Sciences, Mailman School of Public Health, Columbia University, New York, NY USA; 3 Epidemiology group, School of Public Health, Universidad de Antioquia UdeA, Calle 70 No. 52-21, Medellín, Colombia; 4 Department of Community Health and Social Science, City University of New York School of Medicine, New York, NY USA

**Keywords:** Colombia, Health insurance, Cervical Cancer mortality, Policy, Disparities in access

## Abstract

**Background:**

Cervical cancer patients in Colombia have a lower likelihood of survival compared to breast cancer patients. In 1993, Colombia enrolled citizens in one of two health insurance regimes (contributory-private insurance and subsidized- public insurance) with fewer benefits in the subsidized regime. In 2008, the Constitutional Court required the Colombian government to unify services of both regimes by 2012. This study evaluated the impact of this insurance change on cervical cancer mortality before and after 2012.

**Methods:**

We accessed 24,491 cervical cancer mortality records for 2006–2020 from the vital statistics of Colombia’s National Administrative Department of Statistics (DANE). We calculated crude mortality rates by health insurance type and departments (geopolitical division). Changes by department were analyzed by rate differences between 2006 and 2012 and 2013–2020, for each health insurance type. We analyzed trends using join-point regressions by health insurance and the two time-periods.

**Results:**

The contributory regime (private insurance) exhibited a significant decline in cervical cancer mortality from 2006 to 2012, characterized by a noteworthy average annual percentage change (AAPC) of -3.27% (*P* = 0.02; 95% CI [-5.81, -0.65]), followed by a marginal non-significant increase from 2013 to 2020 (AAPC 0.08%; *P* = 0.92; 95% CI [-1.63, 1.82]). In the subsidized regime (public insurance), there is a non-significant decrease in mortality between 2006 and 2012 (AAPC − 0.29%; *P* = 0.76; 95% CI [-2.17, 1.62]), followed by a significant increase from 2013 to 2020 (AAPC of 2.28%; *P* < 0.001; 95% CI [1.21, 3.36]). Examining departments from 2013 to 2020 versus 2006 to 2012, the subsidized regime showed fewer cervical cancer-related deaths in 5 out of 32 departments, while 6 departments had higher mortality. In 21 departments, mortality rates remained similar between both regimes.

**Conclusion:**

Improvement of health benefits of the subsidized regime did not show a positive impact on cervical cancer mortality in women enrolled in this health insurance scheme, possibly due to unresolved administrative and socioeconomic barriers that hinder access to quality cancer screening and treatment.

## Introduction

Globally, in 2019, cervical cancer accounted for between 6.19 and 8.14 per 100,000 deaths among women [1], making it their fourth cause for cancer-related mortality [2]. The significant impact of cervical cancer deaths on women is especially pronounced in low-and-middle-income countries (LMICs), who account for 84–90% of global cervical cancer-related deaths [2]. In Colombia, a lower-middle-income country, cervical cancer is the third incident cancer and cause for cancer mortality among women, after breast and colorectal cancers [2]. Moreover, even though cervical cancer is a preventable and treatable disease, in Colombia women with the disease die in a higher proportion compared to breast cancer [2]. A study by Hernandez et al. revealed that time to first treatment is shorter for women with breast cancer as compared to cervical cancer [3]. As a result, Colombian women diagnosed with breast cancer demonstrate a five year survival probability of 81% versus 53% for women with cervical cancer [4]. Reasons for the difference in mortality rates are not well-understood, but multiple barriers to accessing adequate care and the lack of an organized cervical cancer control program could be playing important roles [5]. Some of the barriers reported are excessive wait-times for service authorizations [6, 7], lack of oncologists, lack of care standardization, and inability to access reliable and affordable transportation within rural areas are other barriers at play [8, 9]. 

The Colombian Political Constitution of 1991 included healthcare as a protected right to all citizens. Until then, most of the population did not have access to health care and there were inequities and inefficiencies in the delivery of health care [10]. By introducing healthcare as a fundamental right, in 1993, Law 100 was established to distribute health care and resources across the country [11]. Colombian healthcare coverage was then provided through two main health insurance systems (contributory-private and subsidized-public) [11]. The “contributory regime” enrolled those who work and pay for insurance [11]. On the other hand, the “subsidized regime” enrolled populations that were unable to pay and therefore their care was provided completely by the State with fewer benefits [11]. Every year, hundreds of demands were presented to the State by citizens who their right to health was being violated. Given this inequality, in 2008, the Constitutional Court of Colombia ordered the government to guarantee equitable and effective universal rights to health through Ruling T760 by equal access to the same quality of care, despite one’s health regime affiliation by July of 2012 [12]. The changes to the subsidized regime included, but were not limited to, more than 2,000 medicines, procedures and health services which they previously had no access to, access to specialized consultation of all kinds, diagnostic tests, and continuity in diagnosis and treatment [12]. Furthermore, the subsidized regime patients were given access to first-time consultations with specialists, without the need for authorization from the Health Secretariats and the paperwork in municipal entities for other types of care was eliminated [12]. 

As of 2022, in Colombia, 98% of the population has health insurance coverage (44.9% contributory and 54.8% subsidized) and regardless of their type of insurance everyone should have the same services [13]. However, this has not guaranteed that all insured individuals have the same access to care. To demonstrate this, around 60% of Colombian women, in the subsidized regime are diagnosed in stages III to IV, whilst only 42% from the contributory regime are diagnosed at those stages [14]. This disparity in diagnosis may be linked to the fact that, only 52% of Colombians in the subsidized regime are able to access primary healthcare services, including screening, compared to 58% of those in the contributory regime [15]. Furthermore, only 55% of those in the subsidized regime obtain an appointment for treatment within the first twenty-three days of diagnosis compared to 65% of women in the contributory regime [16]. Thus, although there is high health insurance enrollment, real-time access to healthcare services is seemingly different based on one’s health insurance [15, 16]. In lieu of that information, we conducted this study to evaluate the impact of the 2012 policy on cervical cancer mortality in Colombia by comparing data from 2006 to 2012 when there were differential services to data from 2013 to 2020, when all women had the same access.

## Materials and methods

### Study design and data sources

In this retrospective study, we compared crude cervical cancer mortality rates between two time periods (2006–2012 and 2013–2020). The original de-identified mortality dataset was made publicly available by the Colombian National Administrative Department of Statistics (DANE) and included 3,439,098 all-cause mortality cases [17]. The original dataset included 35 variables such as age, sex, cause of death, time of death (month, year, hour), country and permanent area or department of residence, educational level, and occupation among others [17]. From these, 10 variables were selected for the final dataset. These variables included: basic cause of death, department of residence, educational level, marital status, age, race/ethnicity, health insurance regime, year of death, and sex. The variables were selected so that we could compare key sociodemographic variables in relation to cervical cancer mortality in each health regime. The other variables did not contain data that helped to answer our objective.

Mortality data, within DANE’s is obtained from death certificates [18]. The information in the death certificates and the overall data are subjected to a Statistical Quality Assurance check [18]. To determine the level of compliance, confidence, and transparency in the statistics generated, a five stage statistical process occurs: detection and analysis of requirements, design and tests, execution, analysis, and dissemination of results [18]. Lastly, DANE implements “Continuous Improvement Requirements” that examin the implementation of and improvement processes of DANE’s statistical process [18]. This involves standardization, quality assurance, including routine audits, to promote uniformity and identify trends [18]. Additionally, proficiency among data collection staff is ensured through capacity-building initiatives [18]. 

Data for the total population at risk, by year, was also obtained. This data came from the census projections made publicly available by DANE [19]. Data on population distribution, by insurance regime and year, was also obtained from publicly available data of the “Report of affiliates by department of the Administrator of the Resources of the General System of Social Security in Health.” [20] However, this dataset lacks information of health regimes by age and sex, making age standardization of rates not feasible in our study.

### Data management

**Fig. 1:**
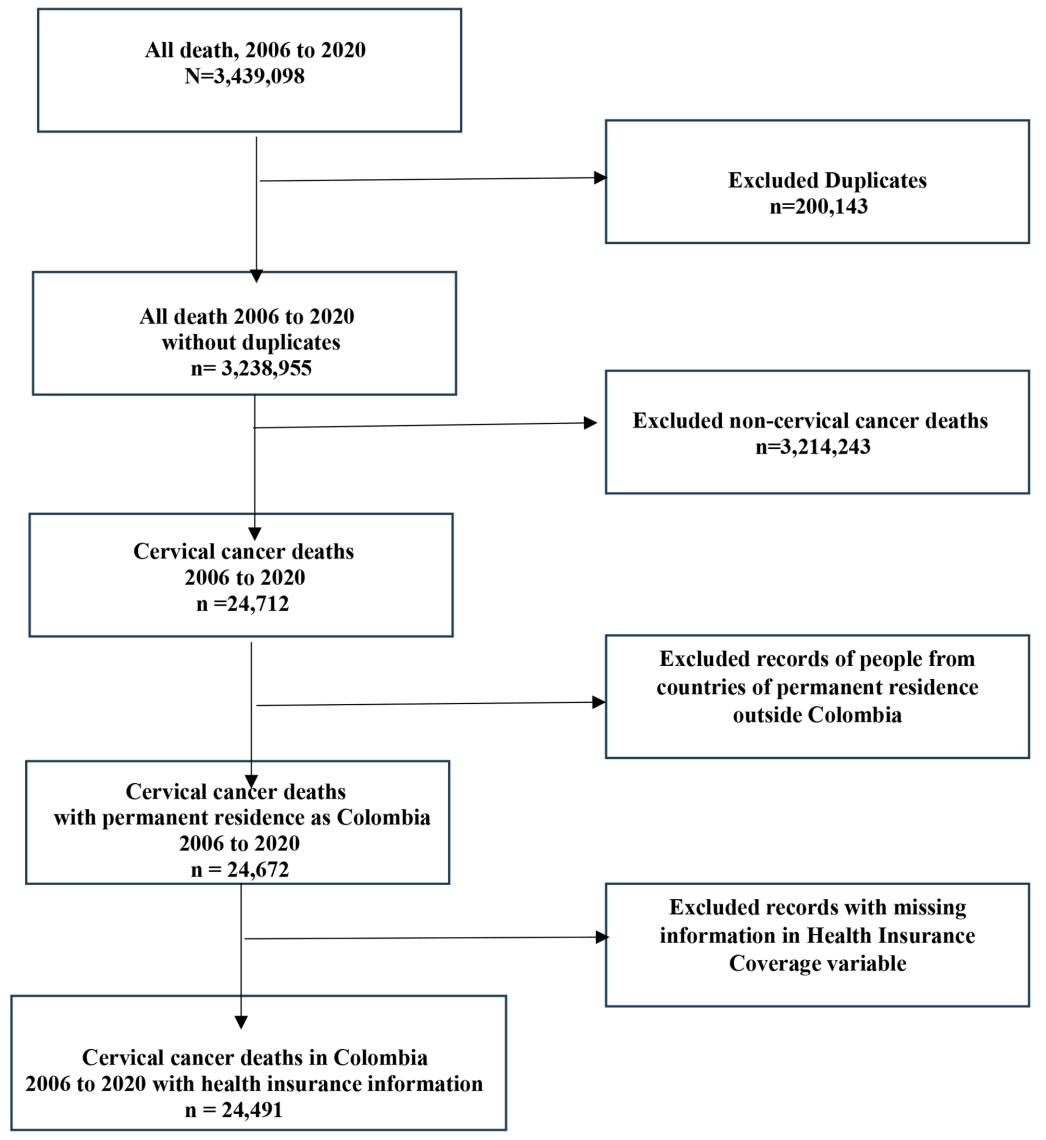
Data Management of DANE mortality data from 2006 to 2020 in Colombia. How data was organized (managed), from the all-mortality Death dataset to the final dataset used for this study. The original dataset had 3,439,098 all-mortality cases and the final dataset had 24,491 cervical cancer mortality cases

Mortality data has a different dataset for each year. Some of the years presented different categories within the variables. Therefore, before merging all years we homogenized the categories within chosen variables across the years. Moreover, all duplicated records were discarded and the ICD10 codes C53.0, C53.1, C53.8, and C53.9 were selected from the variable “basic cause of death”. Finally, we excluded cases with missing data from the variable “health insurance regime” because the researchers were solely interested in making mortality comparisons between those who had one of the insurance plans (subsidized and contributory) or were uninsured. These decisions resulted in a final sample size of 24,491 cervical cancer cases (Fig. 1).

Items in the health insurance variable were recategorized from contributory, subsidized, v*inculado*, *particular*, other, unknown and no information to: contributory, subsidized, uninsured: *vinculado* and *particular*, other, and no information. Items in marital status variable were also reorganized based on the similarity of certain items. For instance, the married and common law unions were combined. Education level and race/ethnicity were also recategorized based on similarity of items. For the variable department of residency “Amazonas, Vaupés, Vichada” were grouped together, due to the low number of inhabitants and therefore cancer cases. Consequently, to ensure rate stability, these departments are collectively analyzed as a single group—a method employed by the Colombian National Cancer Institute in calculating geographic rates [21]. 

### Statistical analysis

Socio-demographic variables were analyzed by health insurance type using chi-squared. Crude cervical cancer mortality from 2006 to 2020 was calculated by year. After, crude cervical cancer mortality rates by insurance regime and year were calculated (total # of cervical cancer deaths in regime w in year x) / (total female population in regime w in year x) *100,000. We had access to the number of Colombians in each regime, but it was not categorized by sex; therefore, based on census data from 2006 to 2020 that reports that 52% of Colombians were women, we used this percentage to estimate the number of women enrolled in each regime. This approach introduces potential limitations, as changes in population composition or enrollment patterns over time may impact the precision of our estimates. It is crucial to recognize and account for these limitations when extrapolating gender distribution from census data, as deviations from actual figures within each regime may occur.

Next, trends in cervical cancer mortality rate by insurance type and time-period were calculated through Joinpoint regressions. Average Annual percentage changes (AAPC) were used to measure increased or decreased mortality from one time-period to the next. Statistical significance was determined by *p* < 0.05. Please note that the results from the category of “other” from the health insurance variable are not presented since we could not determine an accurate denominator. Additionally, cervical cancer mortality rates by department, health insurance regime and year were calculated: (total # of deaths from cervical cancer in women from department z, in regime w and year x) / (total female population from department z, in regime w and year x) *100,000.

To determine the presence and magnitude of gaps between the contributory and subsidized insurance regimes within each period, we calculated the difference of departmental rates: (*crude mortality in the subsidized regime from 2006 to 2012 in department z)–* (*crude mortality in the contributory regime from 2006 to 2012 in department z*) and (*crude mortality in the subsidized regime from 2013 to 2020 department z)–* (*crude mortality in the contributory regime from 2013 to 2020 department z*). Negative results denoted that the subsidized regime had fewer deaths than the contributory regime, whereas positive results represented more deaths in the subsidized regime. Zero represented no gap and higher numbers represented higher gaps between both regimes.

Then, to determine presence and magnitude of gaps between the contributory and subsidized regimes after the inception of Ruling T760, we calculated the difference by department in mortality rates amongst regimes between 2006 and 2012 and 2013–2020: *(crude mortality difference between contributory and subsidized regime from 2013 to 2020 in department z)– (crude mortality difference between contributory and subsidized regime from 2006 to 2012 in department z).* The overall difference between the two periods in each department was represented in a QGIS map. Researchers further identified the regions within which various departments fell and discussed the possible impacts of urban versus rural residence. The positive numbers (darker colors) mean that 2013–2020 had more cervical cancer-related deaths in the subsidized regime compared to the contributory regime. The negative numbers (lighter colors) mean that 2013–2020 had fewer cervical cancer-related deaths in the subsidized regime compared to the contributory regime.

Excel v.2307, IBM SPSS Statistics 29.0.0.0, JoinPoint v 5.0.2 [22], and QGIS v 3.16.16 [23] were used to analyze data. This study used anonymous, secondary mortality microdata from 2006 to 2020 from DANE. Boston University School of Public Health and Universidad de Antioquia did not require an ethics board review for research using anonymous secondary data.

## Results

### Sociodemographic characteristics

Table 1 provides characteristics of all 24,491 cervical cancer mortality cases included in the study, stratified by health insurance regime. Overall, majority of women belonged to the subsidized regime (61%), followed by contributory regime (31%), other (4%) and the uninsured (4%). In all regimes, the women were predominantly between 45 and 64 years old, of mixed ethnicity, lived in a principal municipality, only completed primary education, and were married or in a common law union. All the variables showed statistically significant differences between insurance schemes. For example, the subsidized regime had a higher representation of indigenous peoples (2.5%) and Afro-Colombians (4.8%), followed by women in the contributory regime (0.4% and 3.3% respectively), and uninsured women (0.1%) and 2.5% respectively). In terms of permanent residence, the higher proportion of women living in rural areas were in the subsidized regime (6.3%), followed by uninsured women (3.6%) and the contributory regime (1.4%). Education-wise, more women in the contributory regime had completed secondary school (13.8%), followed by uninsured women (8.6%) and women in the subsidized regime (5.3%). Lastly, more women in the contributory regime (39.1%) were in a common-law union or married, followed by women in the subsidized regime (34.6%) and uninsured women (30.4%).

**Table 1:** Socio-demographic information of 24,491 cervical cancer mortality cases between 2006 and 2020 by health insurance type in Colombia

Variable	Total 24491 (100)	Contributory 7,692 (31.4)	Subsidized 14,878 (60.7)	Other 922 (3.8)	Uninsured 999 (4.1)	*p value*
	N (%)	N (%)	N (%)	N (%)	N (%)	
**Age (in years)**						
5 to 14 15 to 44 45 to 64 64 & above	3 (0) 5162 (21.1) 10457 (42.7) 8857 (36.2)	0 (0) 1611(20.9) 3192 (41.5) 2886 (37.5)	2 (0) 3091 (20.8) 6466 (43.5) 5311(35.7)	0 (0) 270 (29.3) 385 (41.8) 266 (28.9)	1 (0.1) 190 (19.0) 414 (41.4) 394 (39.4)	***<0.001***
**Race or Ethnicity**						
Indigenous Rom, Raizal Afro-Colombian None of the above (Mixed)	426 (1.7) 34 (0.1) 1048 (4.3) 18749 (76.6)	28 (0.4) 18 (0.2) 252 (3.3) 6142 (79.8)	372 (2.5) 15 (0.1) 718 (4.8) 11384 (76.5)	25 (2.7) 0 (0) 53 (5.7) 678 (73.5)	1 (0.1) 1 (0.1) 25 (2.5) 545 (54.6)	***<0.001***
**Permanent residence**						
Principal Municipalities Urban Centre Rural	22680 (92.6) 663 (2.7) 1132 (4.6)	7514 (97.7) 72 (0.9) 105 (1.4)	13385 (90.0) 537 (3.6) 941 (6.3)	846 (91.8) 26 (2.8) 50 (5.4)	935 (93.6) 28 (2.8) 36 (3.6)	***< 0.001***
**Education Level**						
Preschool or No Education Primary Secondary University Education Graduate studies	6990 (28.5) 10571 (43.2) 1989 (8.1) 123 (0.5) 82 (0.3)	1911 (24.8) 3260 (42.4) 1058 (13.8) 76 (1.0) 52 (0.7)	4537 (30.5) 6608 (44.4) 783 (5.3) 27 (0.2) 2 (0)	267 (29.0) 358 (38.8) 62 (6.7) 3 (0.3) 2 (0.2)	275 (27.5) 345 (34.5) 86 (8.6) 17 (1.7) 26 (2.6)	***< 0.001***
**Marital Status**						
Common-law union or Married Single Separated, Divorced, Widowed	8761 (35.8) 4563 (18.6) 5890 (24.0)	3005 (39.1) 1295 (16.8) 1972 (25.6)	5152 (34.6) 2974 (20.0) 3516 (23.6)	300 (32.5) 227 (24.6) 225 (24.4)	304 (30.4) 67 (6.7) 177 (17.7)	***< 0.001***

Table 1 *The *p*-values provided describe the level of significance for the comparisons between the descriptive variables of interest and mortality within health regimes. Missing values: Age 12, Race 4234, Permanent Residence 16, Education level 4736, Marital Status 5277. The People of “Raizal” ethnicity are from the archipelago of San Andres and Providencia. The Principal Municipalities are: Barranquilla, Bogotá, Cali, Cartagena and Medellin.

### Trends by health insurance regime

**Graph 1:**
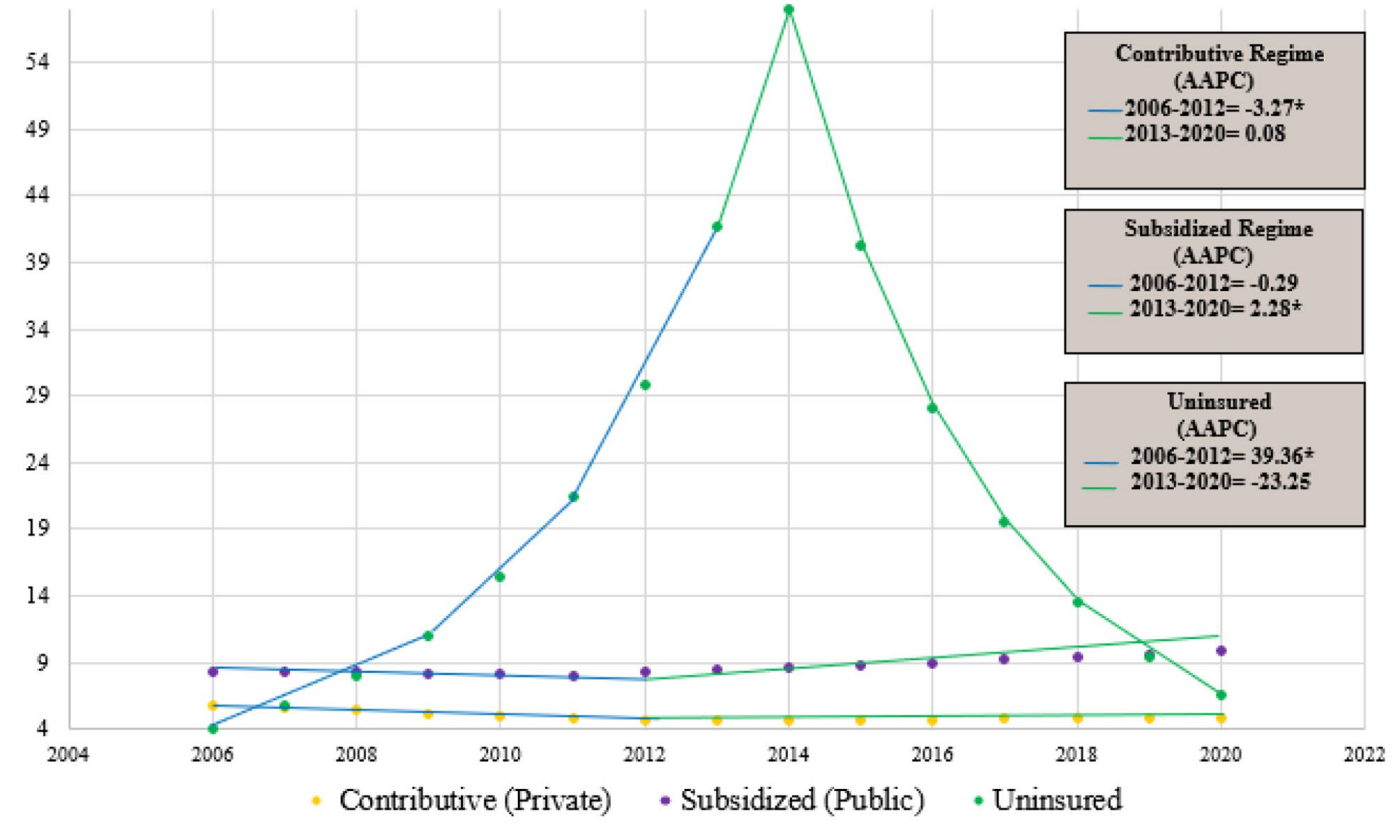
Crude Cervical Cancer Mortality Trends by health insurance status from 2006 to 2020 in Colombia. Graph 1: *Indicates that the AAPC is significantly different from zero at alpha = 0.05. ∼ If the Average Annual Percentage Change (AAPC) is within one segment, the t-distribution is used. Otherwise, the normal (z) distribution is used

Crude mortality in the contributory regime decreased from 5.75 deaths per 100,000 women in 2006, to 4.68 deaths per 100,000 women in 2012. This represented a statistically significant reduction in cervical cancer mortality with an average annual percentage change (AAPC) of -3.27% (*P* = 0.02; 95% CI [-5.81, -0.65]) (Graph 1). Then, from 2013 to 2020 there was a non-statistically significant increase from 4.55/100.000 to 4.97/100.000 (AAPC 0.08%; *P* = 0.92; 95% CI [ -1.63,1.82]).

Comparatively, crude mortality in women in the subsidized regime was 8.45/100,000 in 2006 compared to 8.00/100,000 in 2012, with a non-statistically significant AAPC of -0.29% (*P* = 0.76; 95% CI [ -2.17, 1.62]) (Graph 1). Contrary to our expectations and the intent of the health-regime-benefit-unification policy, from 2013 to 2020, there was a statistically significant increase from 8.13/100,000 to 9.12/100,000 with an AAPC of 2.28% (P = < 0.001; 95% CI [1.21,3.36]).

The uninsured population had a statistically significant increase in mortality rates from 2006 to 2012 (AAPC = 39.36%; *P* = 0.05; 95% CI [ 0.68, 92.89]) (Crude rate: 9.75/100,000 versus 119.80/100,000) (Graph 1). However, from 2013 to 2020, there was a non-statistically significant reduction in crude mortality with an AAPC of -23.25% (*P* = 0.17; 95% CI [-47.61, 12.45]) (Crude rate: 39.47/100,000 versus 10.20/100,000).

### Mortality by department

**Map 1:**
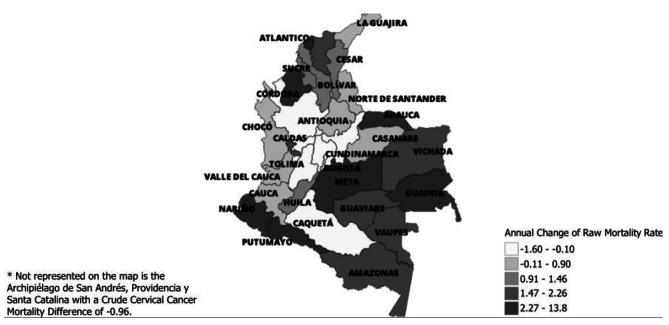
Crude Cervical Cancer Mortality Difference Between Subsidized Regime Compared to the Contributory Regime, from 2006 to 2012 versus 2013 to 2020, in Colombia’s 32 Departments. Map 1: Due to limited space, all department names are not featured on the map. The positive numbers (darker colors) mean that compared to the period 2006–2012, the period 2013–2020 had more cervical cancer-related deaths in the subsidized regime compared to the contributory regime. The negative numbers (lighter colors) mean that, compared to the period 2006–2012, the period 2013–2020 had fewer cervical cancer-related deaths in the subsidized regime compared to the contributory regime

Map 1 focuses on the impact of the 2012 health-regime-benefit-unification policy on cervical cancer mortality by department. Cauca (1.59 fewer deaths/100,000), Caldas (1.07 fewer deaths/100,000), Caquetá (0.81/100,000 fewer deaths), Antioquia (0.76 fewer deaths/100,000), and Chocó (0.62 fewer deaths/100,000 had fewer cervical cancer-related deaths in the subsidized regime compared to the contributory regime- in the period of 2013–2020 in contrast with 2006 to 2012. On the other hand, Cundinamarca (13.84 more deaths/100,000), Norte de Santander (9.57 more deaths/100,000), Archipiélago de San Andrés, Providencia y Santa Catalina (2.94 more deaths/100,000), Nariño (2.53 more deaths/100,000), Casanare (2.31 more deaths/100,000), and Guaviare (2.11 more deaths/100,000) had more cervical cancer-related deaths in the subsidized regime compared to the contributory regime- in the period of 2013–2020 in contrast with 2006 to 2012.

Lastly, Bolívar (0.35 fewer deaths/100,000), Risaralda (0.12 fewer deaths/100,000), Valle del Cauca (0.09 fewer deaths/100,000) and Huila (0.01 fewer deaths/100,000) had a mortality difference of 0.5 or less so they were deemed to have no gaps in mortality. These numbers suggest that cervical cancer mortality, in these departments, remained constant after Ruling T760 (2006–2012).

## Discussion

This study revealed the following interesting observations. First, the contributory regime showed a non-statistically significant increase in crude cervical cancer mortality rates after 2012, whereas the subsidized regime showed a statistically significant increase. Second, after 2012, the uninsured population showcased a non-statistically significant reduction in crude mortality. Lastly, in the period of 2013–2020 in contrast with 2006–2012, six out of 32 departments had higher cervical cancer-related deaths in women enrolled in the subsidized regime compared to those in the contributory regime, whereas five departments had fewer cervical cancer-related deaths in women enrolled in the subsidized regime compared to those in the contributory regime.

The disparity seen between the contributory and subsidized regime after 2012 could be explained by various factors. First, our data reveals higher proportions of mixed ethnicity (79.8%) and secondary school completion (13.8%) among women in the contributory regime, with fewer residing in rural areas (1.4%), which among other factors may be playing a role. Research by Chande et al. indicates that, despite mixed populations exhibiting higher correlations with certain cancers, their higher levels of human development (*R* = 0.60) compared to Afro-Colombians (*R* = 0.20) and Indigenous populations (*R* = 0.59), contribute to better health and cancer outcomes despite a higher risk of disease [24]. More specifically, the risk of dying especially from cervical cancer in Columbia, is significantly higher among primary level educated women (RR = 1.49, *p* < 0.0001) compared to those who completed secondary education (RR = 1.22, *p* < 0.0001) [25]. Considering that socioeconomic status influences educational attainment and, consequently, cervical cancer screening awareness and adherence, the lower mortality rates in the contributory regime after 2012 may be attributed to a combination of these factors promoting better health outcomes and reduced cancer risks in this population.

The urban residency of many women in the contributory regime is another crucial consideration. Majority of cervical cancer care facilities in Colombia are located in urban areas, particularly in the densely populated Andean region, where 50% of the country’s cancer care facilities are situated [26]. As a result, women in the contributory regime have greater access to quality and timely cervical cancer screening and treatment facilities, particularly in urban areas with optimal public transportation [10, 27]. Also, existing literature suggests that women in the contributory regime are less likely to face administrative or socioeconomic barriers to quality cancer care [28]. Literature reports shorter administrative-approval wait times for women in the contributory regime at privately-owned, higher-quality cancer care facilities, further increasing the timeliness of care [29]. Additionally, a study by Chayo et al. [30] reported that women in the contributory regime had a higher (57.9%) five-year overall rate of surviving cervical cancer, compared to 41.9% of women in the subsidized regime simply by receiving timely treatment. The five-year survival rates cement the importance of addressing socioeconomic and accessibility factors in formulating effective policies for cervical cancer prevention and care.

The correlation between private insurance, higher socioeconomic status, and increased chances for cervical cancer survival may not only be applicable in Colombia; evidence of this phenomenon is also observed in other LIMCs and the United States [31–33]. In various LIMCs and the U.S., individuals with higher socioeconomic status encounter fewer obstacles in accessing timely cervical cancer care [31–33]. Lima, Peru serves as an example of this pattern in LIMCs, where urban women, typically covered by private insurance, benefit from enhanced access to screening and specialized cervical cancer care, contributing to increased survival rates [31]. Similarly, in the United States, disparities in insurance coverage play a role, with 70% of white women possessing private insurance, making them 89% more likely to receive prompt early-stage cancer treatment and radiation completion compared to their counterparts, mainly Black or Hispanic women with Medicaid (public insurance) [32]. Consequently, lower five-year cervical cancer survival rates are observed among Black and Hispanic women, who often have public insurance and are more likely to come from lower socioeconomic backgrounds [33]. These findings further underscore the importance of socioeconomic factors in influencing cervical cancer outcomes, in Colombia and in other countries.

In contrast, the statistically significant increase in mortality in women in the subsidized regime, after 2012, observed in our study coincides with multiple reports in the literature that, individuals in this regime are more likely to be impacted by administrative or socioeconomic barriers to quality cancer care [27, 28, 34]. According to our data, a higher proportion of women in the subsidized insurance (6.3%) lived in rural areas. Moreover, a lower percentage of women in the subsidized regime completed secondary school (5.3%). Rural regions are often inhabited by Afro-Colombians and indigenous communities, experiencing internal armed conflict, inadequate healthcare infrastructure, and underdevelopment [35]. Moreover, challenges in accessing transportation, coupled with cultural or language barriers, further diminish the likelihood of survival from cervical cancer in rural areas [26, 35]. 

Transportation expenses from rural areas to urban cancer care facilities pose a financial challenge for subsidized women, often competing with essential household expenses [27–29]. Typically, 6% of Colombian households face difficulties in accessing any healthcare services; however that figure rises to 19% in the poorest rural communities [36]. Consequently, the ability to attend cervical cancer screening and treatment appointments is impeded [7, 26, 28]. This shortage is exacerbated by the absence of oncology instruction in Colombia’s National Cancer Institute’s (NCI) physician training program and medical schools [8, 26]. According to Sardi et al., the NCI annually trains only four gynecological oncologists, two surgical oncologists, and two medical oncologists [34]. As a result, the accessibility of an oncologist is sometimes contingent on one’s ability to seek care at a private health facility, placing women in the subsidized regime at a disadvantage [27]. 

Another significant barrier, that is seldomly considered, is the presence of cultural or language barriers contributing to delayed health-seeking of cervical cancer screening or treatment [28, 37]. Our data reveals a higher percentage of indigenous (2.5%) and Afro-Colombian (4.8%) women under the subsidized regime compared to the contributory regime. Notably, many indigenous populations lack proficiency in Spanish, making adherence to cervical screening programs challenging and leading to a substantial portion of the indigenous population remaining untested [28, 35]. For example, the Amazon region, densely populated by indigenous communities, reports that 20% of women have never undergone a Pap test in their lifetime, compared to the national average of 12.7%.^28^

Similar to rural Colombia, ethnic minorities in rural Brazil encounter challenges in accessing screening tests and cervical cancer treatment due to inadequate insurance benefits, language barriers, transportation costs to urban areas, and a shortage of trained oncological staff in rural facilities [38]. Rural Peruvian women, characterized by lower socioeconomic status, further exemplify the impact of socioeconomic factors and insurance on cervical cancer treatment access [31]. Additionally, rural areas in Nigeria, South Africa, and Uganda also face challenges associated with inadequate rural road systems and unpredictable or inconvenient transportation schedules, making it difficult for women to attend cervical cancer screening appointments [7]. Lastly, in the United States, Black, Hispanic, and American Indian or Alaska Native women concentrated in rural regions exhibit a higher likelihood, compared to White women, of being diagnosed and succumbing to advanced-stage cervical cancer, despite advancements in cervical cancer prevention, screening, and treatment in recent decades [37]. Moreover, these populations, more likely to rely on Medicaid or be uninsured, often encounter difficulties accessing early screenings and experience delays in obtaining further diagnostic testing [37]. 

Overall, the lack of access to transportation, limited number of physicians and the existence of cultural/language barriers, compounded by lower socioeconomic status appear to be some of the disadvantages attributing to less than 15% of subsidized-affiliated Colombians, receiving treatment within 30 days of a cervical cancer diagnosis [28, 29]. Thus, despite efforts towards universalizing health insurance benefits, with Ruling T760, there remains a critical need for comprehensive infrastructure development to overcome all the barriers listed above. Our findings emphasize the urgency of comprehensive policy interventions that go beyond insurance coverage, ensuring that all women, regardless of their socioeconomic status, can effectively navigate and benefit from the healthcare system.

We also observed a significant increase in cervical cancer mortality among the uninsured population from 2006 to 2012. The uninsured demographic in Colombia comprises individuals such as the unemployed, informal workers earning less than the minimum wage, and impoverished families that exceed the income threshold for government social benefits under the subsidized regime. This rise in mortality might be attributed, in part, to the limited access to a comprehensive and high-quality continuum of cancer care [39]. Notably, the two-year overall survival for the uninsured is 52.6%, compared to 64.4% for women with cervical cancer in the subsidized regime [14]. The notable surge in uninsured cervical cancer mortality in 2014 demands attention. After this concerning trend, in 2015, Statutory Act No. 1751 was implemented to establish, regulate, and safeguard the fundamental right to health [40]. Before 2015, those without health insurance had trouble getting the medical services they needed since they were not connected to a health insurance provider [41]. As a result, many who were refused medical care turned to suing using “tutela,” an informal judicial system created by the 1991 Constitution to defend fundamental rights [42]. In 2014 alone, 118,281 lawsuits were filed, reflecting the second-highest number since the passage of the 2008 Ruling T760 [42]. This suggests that in 2014, numerous Colombians despite the passage of Ruling T760, including the 2.7 million uninsured population at the time, were possibly being denied critical services due to the lack of health institution authorization [42]. This denial of services may have contributed significantly to the elevated number of uninsured individuals succumbing to cervical cancer in 2014.

Comparatively, there is a significant decrease in mortality from 2013 to 2020. This decline prompts an exploration of factors contributing to this phenomenon. Our hypothesis is that the combined impact of Ruling T760 and Statutory Act No. 1751 might have worked synergistically to diminish barriers to care affecting the uninsured [42]. Additionally, it is plausible that more individuals from the uninsured population transitioned to being insured, either through the subsidized or contributory regime, depending on their position relative to the poverty threshold.

Regarding the final observation, by insurance regime and department, administrative and socioeconomic barriers may also play a role in one’s ability to access quality cervical cancer screening and treatment. From 2013 to 2020, fewer cervical cancer-related deaths occurred in Cauca, Caldas, Caquetá, Antioquia, and Chocó under the subsidized regime compared to the contributory regime.

Antioquia and Caldas, both situated in the Andean region, house most Colombia’s urban centers, concentrating high-quality cancer care facilities [26]. In the 2000s, the absence of standards verification for cancer-related facilities led to an uncontrolled surge in oncology centers in Colombia’s urban areas [26]. Consequently, the Andean region hosts a significant number, with around thirty oncology centers [26]. This concentration of quality oncology a challenge, given the limited supply of Colombian oncologists [26]. For instance, there are only sixty-nine radio-oncologists, mostly at urban facilities [26]. Thus, the influx of providers to the Andean region limits resources for women in other regions [26]. Moreover, larger cities like Medellin benefit from optimal transportation and communication infrastructure, enabling residents to easily move around and make necessary changes to appointments [26]. 

Caquetá, part of the Amazon region, stands among Colombia’s poorest areas, lacking any oncology centers [26, 43]. On average, 6% of Colombian households struggle to access healthcare, with the figure rising to 16% in the Amazon region [26]. A study highlights a correlation between underreporting and cervical cancer mortality, particularly in resource-deficit rural regions [44]. From 2005 to 2008, rural Caquetá recorded about 10 deaths per 100,000, significantly lower than more urban departments [44]. The study identifies underreporting, possibly linked to lower screening program participation in rural, resource-deficit settings [44]. The impression is that underreporting may have occurred in Caquetá, accounting for the reduction in mortality after 2012.

In comparison, from 2013 to 2020, Cundinamarca, Norte de Santander, Archipiélago de San Andrés, Providencia y Santa Catalina, Nariño, Casanare, and Guaviare experienced an increased incidence of cervical cancer-related deaths under the subsidized regime compared to the contributory regime, diverging from the 2006 to 2012 trend. While Cundinamarca and Norte de Santander, situated in the Andean region, were expected to witness a decrease in mortality, urban inequality in these areas, driven by a growing wealth gap, may be contributing to heightened cervical cancer mortality [43]. Notably, Nariño (Pacific region), Guaviare (Amazon region), Casanare (Orinoco region), and Archipiélago de San Andrés, Providencia y Santa Catalina (Caribbean region) exhibit unique challenges. These regions, deeply affected by internal conflict, face precarious healthcare conditions, with up to 19% of households in the Amazon and Pacific regions unable to access any healthcare services [26]. Furthermore, the Amazonian and Pacific regions, along with the Orinoco and Caribbean regions, harbor indigenous populations, posing challenges in quality cervical cancer reaching them due to language barriers and distance from healthcare facilities [26, 38]. Roncancio, Cutter, and Nardoccio [35] highlight the Caribbean region’s higher vulnerability to underdevelopment, potentially impacting access to quality cancer screening and treatment.

Overall, clear, and comprehensive cervical cancer policies, along with necessary resources, are crucial for promoting screening, treatment, and eliminating cervical cancer in Colombia. We recommend the Precision Public Health (PPH) approach, as demonstrated in genomic medicine studies, for targeted interventions based on health demographics [45]. For instance, a study in Chocó identified a gene variation in Afro-Colombians affecting the drug Tacrolimus’s dosage after organ transplants, highlighting the potential for tailored medical interventions [45]. Applying PPH to cervical cancer care involves a thorough assessment of health demographics, including geography, education, socioeconomic status, and cultural backgrounds. Given the higher representation of Afro-Colombians and indigenous populations in the subsidized regime, collaborative interventions should prioritize factors like linguistic accessibility, community involvement, and cultural sensitivity, with active community participation throughout the policy development, implementation, and ongoing evaluation process [28, 37]. 

Leveraging the PPH approach as a cornerstone of government policy, culturally and linguistically competent cervical cancer screening methods indigenous populations, are crucial [28]. With 20% of indigenous women never having undergone a Pap test, exceeding the national average, the limitations of Pap tests and the success of self-collected HPV tests underscore the need for a paradigm shift [28, 46]. Successful interventions in Guatemala and Canada, involving self-collection kits, culturally sensitive information sessions, and engagement with community health workers, achieved high willingness and ease of use among indigenous participants, fostering awareness and identification of HPV [46, 47]. Combining self-collection tests with a multi-level promotion strategy in Colombia, delivered in indigenous languages, has the potential to reduce transportation costs, enhance privacy, and increase awareness, resulting in more effective cervical cancer screening. Another recommendation is the incentivization of high-quality cervical cancer care providers to encourage work in more rural or remote areas [8, 26]. 

There are a few limitations to this study. DANE’s dataset does not report the cancer-stage at diagnosis or death, which would have provided useful information on the likelihood of treatment survival and the effectiveness of existing screening programs. Only five cities, in Colombia, have cancer registries and access to data is limited. Therefore, obtaining cancer-stage at diagnosis or death is difficult. Furthermore, we were unable to calculate age-standardized mortality rates by healthcare insurance regime since we did not have access to data on health insurance enrollment by age and sex. To address this gap, an alternative method that future researchers could use is statistical imputation techniques that estimate age-standardized mortality rates in the absence of disaggregated data. In addition, we did not do an analysis of the out-of-pocket payers. This was not possible because our data is categorized by contributory (private), subsidized (public)and uninsured. Notably, the uninsured may not always be synonymous with out-of-pocket as Colombians from the contributory scheme may also experience out of pocket expenses. Furthermore, although other studies have been able to calculate out-of-pocket costs, they used privately-owned data sources, that we do not have access to. Therefore, we cannot make such an analysis in our paper. Nonetheless, out of pocket payments should be considered in future studies, using the DANE database or other databases, as that data becomes available. Moreover, another limitation of the study is the proposed time frame of 2013–2020. Seven years may not be sufficient to capture the full impact of the law. Unfortunately, the DANE dataset does not extend beyond 2021. Therefore, future research should extend the time frame of mortality data, as DANE or other sources add more years to their databases.

Despite its limitations, this study is the first to examine the influence of Ruling T760 on cervical cancer mortality. The findings highlight that despite attempts to guarantee equitable access to quality cervical cancer care services for all Colombians, regardless of health regime affiliation, significant disparities in mortality persist between those in the subsidized regime and those in the contributory regime. Secondly, our study provides a useful framework around which future studies or recommendations can be made. The discussion acknowledges areas of concern, such as the need for comprehensive infrastructure development, addressing socioeconomic disparities, and enhancing healthcare accessibility, underscoring the multifaceted nature of the issue. Therefore, with more complete data, future studies could also explore mortality by age, socioeconomic status, ethnicity, education etc. to explore how these factors impact patient navigation within both health regimes. Another study might also be able to identify specific socioeconomic barriers to access that impact rural versus urban populations. Lastly, future studies could identify the differences between late-stage and early-stage cervical cancer patients with respect to access to care.

## Conclusion

The improvement of health benefits for the subsidized regime, in 2012, appeared not to have a positive impact on cervical cancer mortality. From 2013 to 2020, cervical cancer mortality increased in both the contributory and subsidized regime, with greater mortality in the subsidized regime. Furthermore, only 5 out of 32 departments had fewer cervical cancer-related deaths in the subsidized regime compared to the contributory regime, from 2013 to 2020. Beyond administrative and socioeconomic challenges, the intricate web of ethnic disparities, educational variations, regional geography, and healthcare accessibility emerges as key possible contributors to this unsettling reality. The findings underscore the need for a multifaceted approach, acknowledging the influence of ethnicity, education, and geography on healthcare outcomes. As a result, the study advocates for Precision Public Health, culturally sensitive screening methods, provider incentivization, and comprehensive infrastructure development to possibly address these complex challenges and pave the way for improved cervical cancer outcomes in Colombia.

## Data Availability

The dataset supporting the conclusions of this article are available on Colombia’s National Administrative Department of Statistics (DANE) website, under the Salud (Health) section, https://microdatos.dane.gov.co/index.php/catalog/SAL-Microdatos.

## Abbreviations

CCCMRs: Crude Cervical Cancer Mortality Rates
DANE: Spanish acronym for Colombia’s National Administrative Department of Statistics
LIMCs: Low-and-middle-income countries
HPV: Human papillomavirus
PPH: Precision Public Health
